# Wheelchair service provision education in academia

**DOI:** 10.4102/ajod.v6i0.340

**Published:** 2017-09-08

**Authors:** Karen H. Fung, Paula W. Rushton, Rachel Gartz, Mary Goldberg, Maria L. Toro, Nicky Seymour, Jonathan Pearlman

**Affiliations:** 1 School of Rehabilitation, Université de Montréal, Canada; 2 Marie Enfant Rehabilitation Center, Sainte-Justine University Hospital Research Center, Canada; 3 Rehabilitation Science & Technology, University of Pittsburgh, United States; 4 Human Engineering Research Laboratories, VA Pittsburgh Healthcare System, United States; 5 Department of Physiotherapy, Universidad CES, Colombia; 6 Motivation Charitable Trust, South Africa

## Abstract

**Background:**

An estimated 70 million people with disabilities need wheelchairs. To address this global crisis, the World Health Organization (WHO) proposed an eight-step wheelchair service provision model to ensure service quality regardless of resource setting. The International Society of Wheelchair Professionals (ISWP) aims to facilitate the integration of the WHO eight-step model into professional rehabilitation programmes.

**Objective:**

To develop an enhanced understanding of the current wheelchair service provision education provided in professional rehabilitation programmes worldwide.

**Methods:**

In a cross-sectional design, an online survey was distributed to ISWP contacts of educational institutions. Quantitative responses were analysed through summary statistics and qualitative answers were analysed by content analyses. When relevant, educational institutions were stratified into resource settings.

**Results:**

Seventy-two representatives of educational institutions in 21 countries completed the survey. Wheelchair content was taught in 79% of represented institutions, of which 75% of respondents reported using original course material, 10% of respondents used WHO Wheelchair Service Training Packages and 15% of respondents used other available resources. The majority of educational institutions teaching with their own wheelchair-related course material taught ≤ 20 hours. Fourteen of the 15 respondents without wheelchair education, expressed an interest in integrating wheelchair education into their academic curricula.

**Conclusion:**

The majority of the educational institutions teach wheelchair education; however, there is great variability in what and how it is taught and evaluated. The results demonstrate the need for more in-depth investigation regarding the integration process of wheelchair education in educational institutions, with the ultimate goal of improving wheelchair service provision worldwide.

## Introduction

The World Health Organization (WHO) estimates that there are 70 million people worldwide who require a wheelchair for mobility (World Health Organization 2008). According to the Jhpiego Corporation, the percentage of demand met for wheelchairs in low-resourced countries is often below 5% (Jhpiego Corporation 2015). Even for the people who do have wheelchairs, a significant number use poorly fitting or inappropriate wheelchairs, which may lead to secondary injuries and to a high likelihood of abandoning the technology (Jhpiego Corporation 2015). The wheelchair service provider is tasked with providing a wheelchair that meets the needs of the user in relation to the user’s environment and daily activities, which often includes complex postural support and pressure relief.

According to the World Report on Disability, many countries have an unequal geographic distribution of rehabilitation professionals (World Health Organization 2011), and thus, the profession of wheelchair service providers may vary geographically. To accommodate for the lack of rehabilitation professionals in less-resourced settings, the WHO has suggested using existing personnel to deliver wheelchair services, including community healthcare workers, community-based rehabilitation workers, nurses, physical therapists, occupational therapists, orthotists and prosthetists (World Health Organization 2011). Depending upon the profession and the setting, wheelchair service provision education may be provided by non-governmental organisations or by health professional academic programmes, with variations among educational programmes. Indeed, lack of adequate training has been identified as a major factor in the lack of appropriate wheelchair provision in less-resourced settings (World Health Organization 2011) and also high-resourced settings (HRSs) (Fifield & Fifield 1997; Kanny & Anson 1998; Lenker 1998).

University professional programmes in occupational therapy, physical therapy, prosthetics and orthotics are governed by organisations at various levels. For example, occupational therapy programmes at Canadian universities are approved at an international level by the World Federation of Occupational Therapists (2016) and are supported nationally by the Association of Canadian Occupational Therapy University Programs (2016) who work in conjunction with the Canadian Association of Occupational Therapists (2012) to achieve and uphold education standards. Scope of practice is determined by provincial acts and guided in part by the Profile of Occupational Therapy Practice in Canada (Canadian Association of Occupational Therapists 2012).

The need to navigate organisations at various levels when developing curricula is similar across occupational therapy, physical therapy and orthotics and prosthetics and scope of practice regarding a profession’s role in the wheelchair service delivery process is often influenced by the geographic location of the university. One of the challenges with respect to curriculum development is the scope of each of these professions, where wheelchair service delivery is only one of many content areas that need to be included within the university programmes. Even within occupational therapy alone, the inclusion of wheelchair content in curricula is mandated in some countries (e.g. the United States), but not in others (e.g. Canada).

The recent WHO recommendation of an eight-step wheelchair service provision process (World Health Organization 2008) has the potential to guide university curriculum development in this area of practice. The eight steps, including (1) referral and appointment, (2) assessment, (3) prescription, (4) funding and ordering, (5) product preparation, (6) fitting, (7) user training and (8) maintenance, repairs and follow-up, were developed to ensure appropriate wheelchair service provision to any person in any setting. The WHO has subsequently developed Wheelchair Service Training Packages (WHO WSTP) at the basic, intermediate, manager and stakeholder levels, of which various components are available in multiple languages (World Health Organization 2012, 2013, 2015). These packages include open-access training materials with resources such as training manuals, participant workbooks, presentations, videos and posters. Use of the WHO eight-step wheelchair service provision model has demonstrated positive outcomes (Toro, Eke & Pearlman 2016).

Other resources may be used to complement the WHO’s eight-step process, including the Rehabilitation Engineering & Assistive Technology Society of North America practice guidelines that reflect these eight steps (Arledge et al. 2011), the Wheelchair Skills Program, which focuses on wheelchair skills testing and training (components of steps 2 and 7) (Kirby et al. 2016a) and the Wheelchair Maintenance Program (steps 7 and 8) (Toro et al. 2017).

Recognising the multifaceted challenges associated with integrating new content into academic curricula (i.e. the WHO eight-step wheelchair service provision model), the International Society of Wheelchair Professionals (ISWP 2016) has formed a committee dedicated to supporting the integration of wheelchair service provision content into educational programmes across high- and low-resourced settings (LRSs). The ultimate goal is to ensure that everyone who needs a wheelchair receives an appropriate one and is trained to use it and maintain it. Increasing the number of professionals trained in appropriate wheelchair service provision will help to achieve this goal. At present, there is a paucity of knowledge regarding education provided in curricula in this area of practice. As a first step towards accomplishing this goal, the objective of this study was to describe the current wheelchair service provision education offered in professional rehabilitation programmes in different resource settings across the world.

## Methods

### Design

This project used a cross-sectional survey design, in order to acquire data regarding the current situation in wheelchair service provision education from educational programmes worldwide in a cost-effective manner (Hall 2011). The data were collected as part of a larger study, which surveyed respondents from both educational and non-educational institutions worldwide.

### Recruitment and sample

A geographically diverse convenience sample of respondents was recruited through the ISWP listserv (e.g. individual university contacts and World Confederation for Physical Therapy’s Network for Physical Therapy Educators) and snowball sampling. The invitation to participate and the survey link were sent via email with recruitment beginning on 05 August 2015 and remaining open until 02 September 2015. Respondents were not reimbursed for their time.

### Measurement

The ISWP developed the survey content based on committee members’ knowledge of the wheelchair service provision process. It was formatted using Survey Monkey (www.surveymonkey.com). The final version was based on iterative feedback from committee members and pilot testing of the online version by two committee members. To ensure that responses were based on shared definitions, the survey defined ‘basic’ wheelchair content as including core knowledge and the WHO eight-step model and the ‘intermediate’ level of education was defined as including information beyond the basic level, such as information regarding postural support for wheelchair users and supplementary-advanced wheelchair provision for children (World Health Organization 2012, 2013).

The final survey included 27 questions in total. Respondents were first asked a series of demographic questions (*n* = 9) followed by a question about current wheelchair service provision education (*n* = 1). Depending on the response to this question, each respondent was led to one of three possible sets of questions pertaining to: (1) original wheelchair material (*n* = 5) for those developing and teaching their own content, (2) use of WHO WSTP (*n* = 7) for those using existing materials or resources and (3) interest in teaching wheelchair service provision content (*n* = 5) for those who have not yet integrated wheelchair content into their curriculum. Each set was composed of mandatory and optional questions. Thus, the number of respondents varied per question (i.e. the sum of respondents per question was not equivalent to the number of respondents directed to the set of questions). The response formats included yes or no dichotomous choices (e.g. awareness of WHO WSTP and inclusion of wheelchair service provision content in curriculum), check boxes for lists (e.g. programmes offered in your institution and level of teaching material), typing boxes for individualised responses (e.g. name of institution and time spent teaching wheelchair service education) and large typing boxes for optional qualitative comments for elaboration (e.g. types of wheelchair service education practicum and testing).

### Analyses

Raw data were downloaded from Survey Monkey and exported into Microsoft Excel 2011 (Microsoft Corporation, Redmond, WA). Quantitative responses were combined and summary statistics calculated (when appropriate) using Microsoft Excel 2011. Frequencies were presented as percentages and fractions, such that the denominator represented only the number of respondents who answered each question. Educational institutions were stratified into low income, lower middle income, upper middle income and high income according to the World Bank definitions (The World Bank Group 2016). Respondents from low income and lower middle income countries were collapsed into a ‘low-resourced’ category because of low participation rates in these two categories. Qualitative comments were analysed by frequency for each topic, with the most frequent comments reported in the results as examples.

**FIGURE 1:**
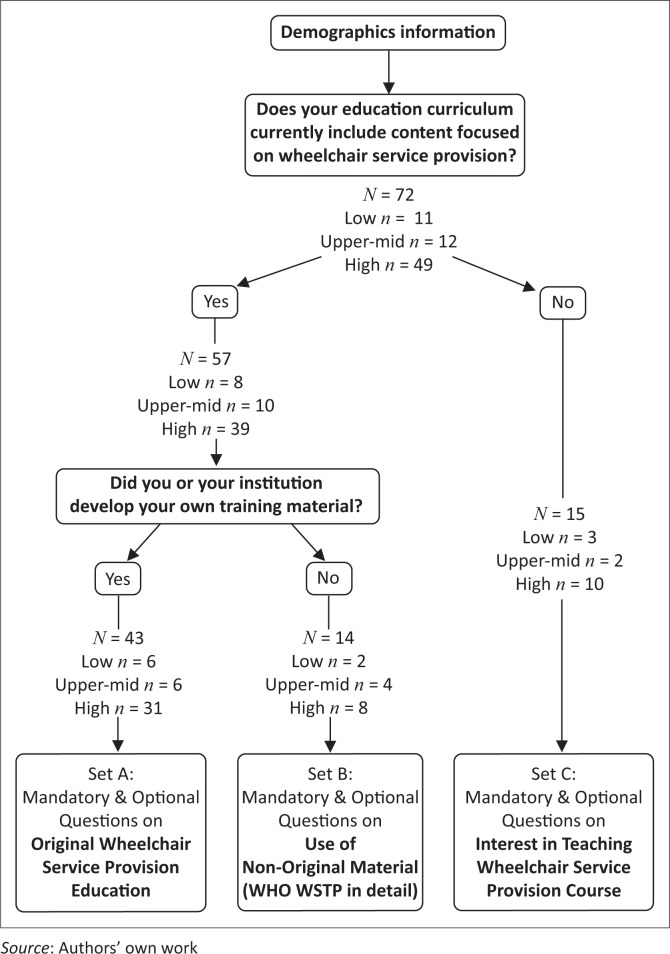
Flow chart of the survey pathway and the sample sizes.

**FIGURE 2:**
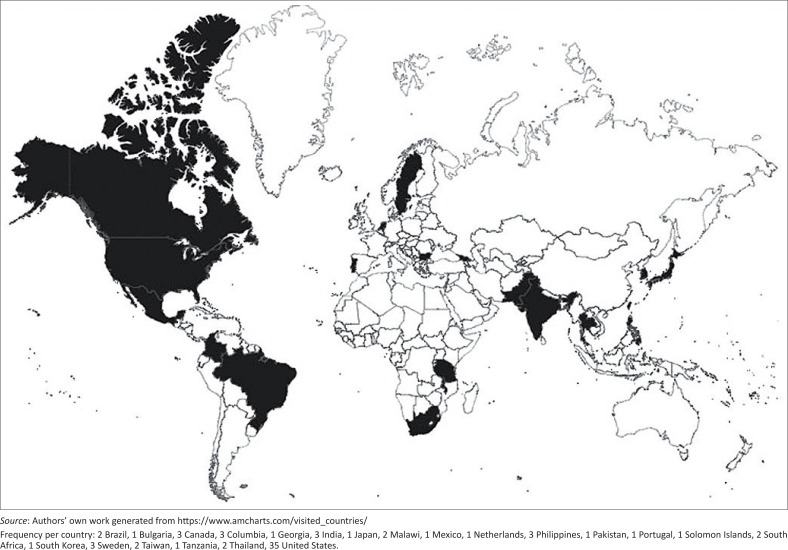
Countries of educational institutions respondents (*n* = 72).

The flow chart of Figure 1 was created with CmapTools Version 6.01.01 (Florida Institute For Human and Machine Cognition, Pensacola, FL). Mapping of the geographic distribution of respondents as shown in Figure 2 was created using amcharts.com. The following page was accessed on 09 May 2016: https://www.amcharts.com/visited_countries/.

### Ethical consideration

This survey was approved through the Institutional Review Board (exempt PRO15060076) at the University of Pittsburgh.

## Results

### Demographic information

A total of 72 representatives from educational institutions responded to the survey (Table 1). The respondents’ progression through the survey consisted of 43, 14 and 15 respondents directed into the set of questions regarding original wheelchair material, use of WHO WSTP and interest in teaching wheelchair service provision content, respectively (Figure 1). Of the respondents, 11/72 (15.3%) were from LRSs, 12/72 (16.7%) from upper middle-resourced settings (UMRSs) and 49/72 (68.1%) from HRSs (Figure 2). The majority of the 72 educational institutions were a university type of institution. In addition to the professional programmes listed in Table 1, other related programmes offered in LRSs and UMRSs included rehabilitation care (*n* = 5) and, in HRSs, occupational therapy assistants (OTAs) programme (*n* = 4).

**TABLE 1:** Professional rehabilitation programmes offered by types of educational institutions and resourced settings.

Variable	*n*	Professional rehabilitation programmes					Teach wheelchair content *n* (%)
		Physical therapy *n* (%)	Occupational therapy *n* (%)	Prosthetics and orthotics *n* (%)	Wheelchair service provision *n* (%)	Other programmes *n* (%)	
University							
LRS	10	6 (60.0)	4 (40.0)	4 (40.0)	3 (30.0)	6 (60.0)	7 (70.0)
UMRS	11	4 (36.4)	5 (45.5)	4 (36.4)	4 (36.4)	5 (54.5)	9 (81.8)
HRS	37	4 (10.8)	34 (91.9)	0 (0.0)	5 (13.5)	2 (16.2)	31 (83.8)
Community or technical college							
LRS	1	0 (0.0)	0 (0.0)	0 (0.0)	1 (100.0)	0 (0.0)	1 (100.0)
UMRS	0	0 (0.0)	0 (0.0)	0 (0.0)	0 (0.0)	0 (0.0)	0 (0.0)
HRS	12	0 (0.0)	10 (83.3)	2 (16.7)	2 (16.7)	4 (33.3)	8 (66.7)
Other type of educational institution							
LRS	0	0 (0.0)	0 (0.0)	0 (0.0)	0 (0.0)	0 (0.0)	0 (0.0)
UMRS	1	1 (100.0)	1 (100.0)	1 (100.0)	1 (100.0)	0 (0.0)	1 (100.0)
HRS	0	0 (0.0)	0 (0.0)	0 (0.0)	0 (0.0)	0 (0.0)	0 (0.0)

**Total**							
**LRS**	**11**	**6 (54.5)**	**4 (36.4)**	**4 (36.4)**	**4 (36.4)**	**6 (54.5)**	**8 (72.7)**
**UMRS**	**12**	**5 (41.7)**	**6 (50.0)**	**5 (41.7)**	**5 (41.7)**	**5 (41.7)**	**10 (83.3)**
**HRS**	**49**	**4 (8.2)**	**44 (89.8)**	**2 (4.1)**	**7 (14.3)**	**6 (12.2)**	**39 (79.6)**

*Source*: Authors’ own work

*n*, number of respondents; LRS, low-resourced setting; UMRS, upper middle-resourced setting; HRS, high-resourced setting.

### Current wheelchair service provision content education provided

The majority of respondents (57/72, 79.2%) reported an incorporation of wheelchair service provision content in their curricula. Regardless of the type of educational institution or resource level, respondents primarily used material developed by their own institutions as part of the teaching methods (43/57, 75.4%) (Table 2). Of the educational institutions that teach wheelchair service provision using the WHO WSTP content, whether they also used original material or not, 3/8 (37.5%) were from LRSs, 3/10 (30%) were from UMRSs and 1/39 (2.56%) was from HRS. Additionally, 2/39 (5.1%) from HRSs indicated the use of the Wheelchair Skills Program (Kirby et al. 2016a) in the ‘others’ answer box and four other respondents also reported doing so in the typing comments boxes at the end of the survey.

**TABLE 2:** Frequency of wheelchair service content taught by resource settings.

Variable	*n*	Original material *n* (%)	WHO WSTP *n* (%)	WSP *n* (%)	Other material *n* (%)
LRS	8	6 (75.0)	3 (37.5)	0 (0.0)	1 (12.5)
UMRS	10	6 (60.0)	3 (30.0)	0 (0.0)	2 (20.0)
HRS	39	31 (79.5)	1(2.6)	2 (5.1)	3 (7.7)

**Total**	**57**	**43 (75.4)**	**7 (12.3)**	**2 (3.5)**	**6 (10.5)**

*Source*: Authors’ own work

*n*, number of respondents; LRS, low-resourced setting; UMRS, upper middle-resourced setting; HRS, high-resourced setting.

#### Original wheelchair service provision content

Of the 43 respondents who reported development of original wheelchair service provision content, 6/8 (75%) were from LRSs, 6/10 (60%) from UMRSs and 31/39 (79.5%) from HRSs. Of the 42/43 (97.7%) responses to the question regarding level of education, it was reported that basic (19/42, 45.2%), intermediate (7/42, 16.7%) and a combination of basic and intermediate (14/42, 33.3%) were taught. One respondent did not answer this question and two respondents provided qualitative information only. Although 10 respondents did not provide a response, 33/43 (76.7%) respondents reported the number of hours spent teaching original wheelchair service provision content. The range per setting was 2–45 h for HRSs (mean: 13 h, standard deviation: 10.1 h) (*n* = 25), 6–32 h for UMRSs (*n* = 4) and 3–35 h for LRSs (*n* = 4). At 27/33 (81.8%) educational institutions, wheelchair service provision content was taught for 20 h or less.

Of 42 responses to the question on pedagogical methods, 28 (66.7%) reported the inclusion of practical sessions. For 3/5 (60%) educational institutions from LRSs and 2/4 (50%) educational institutions from UMRSs, practical involved wheelchair provision to actual wheelchair users, as per qualitative comments. Of the 19 educational institutions from HRSs that responded, wheelchair service provision simulations (6/19, 31.6%), ‘a day in a wheelchair’ (3/19, 15.8%) and wheelchair service provision at a seating clinic (2/19, 10.5%), including an outreach clinic on a mission trip to Haiti, were examples of practical experiences provided via qualitative comments. Thirty-seven of these 42 respondents (88.1%) also reported that their curricula included student evaluations on wheelchair content. For those respondents who elaborated on their testing processes via qualitative comments, it was reported that written (*n* = 17) and practical exams (*n* = 25) were used.

#### World Health Organization Wheelchair Service Training Packages

**FIGURE 3:**
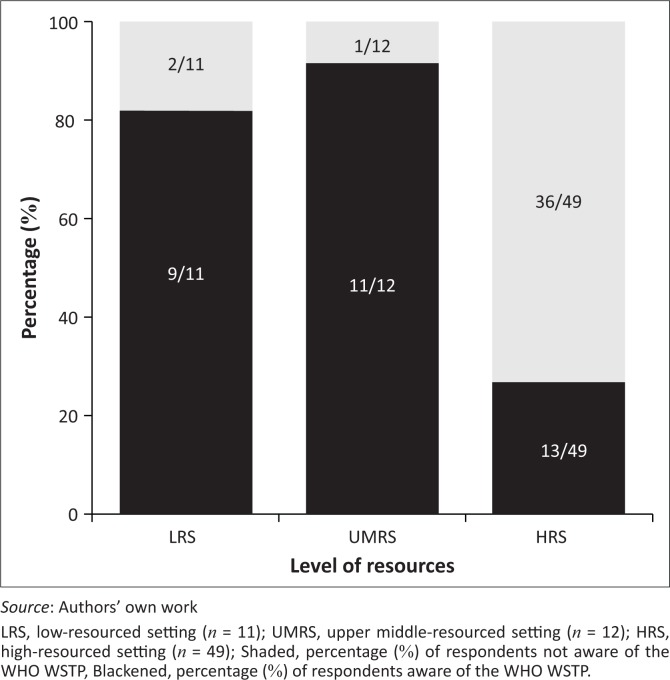
Awareness of World Health Organization Wheelchair Service Training Packages in educational institutions based on level of resources.

Of the 72 survey respondents, 33 (45.8%) were aware of the WHO WSTP, including 9/11 (81.8%) from LRSs, 11/12 (91.7%) from UMRSs and 13/49 (26.5%) from HRSs (Figure 3). Seven of these respondents reported using the WHO WSTP (LRSs: *n* = 3; UMRSs: *n* = 3; HRS: *n* = 1). While 2/7 (28.6%) did not respond, 5/7 (71.4%) provided insight regarding which WHO WSTP packages were used: 4/5 (80%) respondents reported that they used the basic package and 1/5 (20%) (from UMRS) reported that they used both the basic and intermediate packages. The packages were used in their entirety by 4/5 (80%) respondents. In response to the time frame during which the WHO WSTP was taught, 3/4 (75%) respondents taught the basic package in a continuous block, while one respondent taught the basic package throughout the programme. The WHO WSTP was taught by either a professor in the department (2/4, 50%) or a local service provider (2/4, 50%). The WHO WSTP was integrated towards the end of the curriculum for the three respondents who answered this question. Five respondents provided additional comments regarding the universal applicability of the WHO WSTP with the understanding that adaptations may be required to accommodate specific contexts, as recommended in the WHO WSTP.

Comments provided additional insights into respondents’ opinions regarding the WHO WSTP. The respondents from educational institutions that offered an OTA programme commented that many of the tasks in the WHO eight-step model were beyond the role of an OTA. Other respondents (*n* = 2) would like to see wheelchair service provision content integrated into programmes such as community-based rehabilitation work, medics and paramedics. Two additional respondents would like to see the WHO eight-step model adopted as the educational standard by the national accrediting bodies (e.g. Association of Canadian Occupational Therapy University Programs) and by the world federations of rehabilitation professionals (e.g. Federation of Occupational Therapists and World Confederation for Physical Therapy). Finally, three respondents noted their hopes of seeing ISWP change international policies on wheelchair service provision by establishing the WHO eight-step model as the educational standard.

#### Interested in teaching

Fifteen of 72 respondents (20.8%) reported that they did not currently teach wheelchair service provision content, most (10/15, 66.7%) from HRSs. An interest in integrating wheelchair service provision content, however, was expressed by 14 of these 15 respondents (93.3%), 5 (35.7%) of whom were aware of the WHO WSTP as an existing resource. Of these 14 respondents, two participants (14.3%) did not answer the following question, but 5/12 (41.7%) reported that they had previously contacted an organisation or an individual to obtain information on the integration of wheelchair service provision content into their curriculum. Nine respondents predicted that an average of 12.4 ± 12.0 h (range: 1–35 h) could be potentially reserved for wheelchair service provision education at their institutions, where 2/9 (22.2%) were from LRSs, 2/9 (22.2%) from UMRSs and 5/9 (55.5%) from HRSs. Ultimately, 10/12 (83.3%) respondents expressed interest in the WHO WSTP, with four of these respondents specifically interested in integrating WHO WSTP material into their curriculum.

## Discussion

We achieved our goal of developing a more comprehensive understanding of the current state of wheelchair service provision education provided in academic curricula around the world. With responses from 72 educational institutions from 21 countries of all resource settings, this is one of the first studies to investigate this situation on a global scale. This survey expands on previous studies that examined only partial aspects of the wheelchair service provision education offered in professional rehabilitation programme curricula, such as wheelchair assessment and skills training (Best, Miller & Routhier 2015; Coolen et al. 2004; Kirby et al. 2011; White 2003) or wheelchair prescription (Silcox 1995).

Most educational institutions taught original wheelchair service provision education at a basic level, which includes topics from the WHO eight-step model, or content at an intermediate level. However, the commonly reported duration of wheelchair-related education is well under the 35–40 h recommended to teach the WHO eight-step model using the tool of WHO WSTP that was developed by a team of experts around the world and represents the minimum standard from the perspective of WHO. From our findings, the difference in time spent teaching is perhaps an indication that not all topics from the WHO eight-step model were covered in original wheelchair service provision content. Consequently, students receiving training through these programmes may not acquire the knowledge or skills necessary to provide basic wheelchair service.

Most original wheelchair service provision education included practical training and testing developed in-house. In lieu of an institution-developed written test, an alternative could be the ISWP Wheelchair Service Provision – Basic Test, a tool developed and validated by the ISWP to measure the basic competency of wheelchair professionals worldwide (Gartz et al. 2017).

A small portion of education institutions, mostly from LRSs and UMRSs, used the WHO WSTP to teach wheelchair service provision education. One possible reason for the regional bias of WHO WSTP use is that the initial efforts by the WHO targeted LRSs, that is, when it first published the WHO eight-step model for wheelchair service provision (World Health Organization 2008). Additionally, a previously identified barrier in the integration of new topics in rehabilitation programme curricula is the lack of faculty interest and expertise (Kanny, Smith & Dudgeon 2005). In our study, a lack of knowledge was found, such that 36/49 (73.5%) respondents from HRSs were unaware of this resource. To date, the WHO WSTP is the only readily available training tool that focuses on the WHO eight-step model. Building on the survey results, other possible methods to teach the WHO eight-step model are in development, such as the ISWP Hybrid Course that combines online self-study and face-to-face practical training. Additionally, the ISWP has launched an outreach campaign to raise global awareness about quality wheelchair products and services, and the variety of available resources, including the WHO WSTP, to improve the situation. Our results suggest that a new target of these awareness campaigns should be educational institutions in HRSs, while ascertaining the awareness and maintaining the interest of educational institutions from LRSs and UMRSs.

Although not explicitly asked in the survey, the Wheelchair Skills Program was identified as a wheelchair education tool in four curricula in HRSs. The Wheelchair Skills Program is another resource that concentrates on and enhances two steps of the WHO eight-step model: assessment and user training. The Wheelchair Skills Program is shown to be efficient in different contexts in the world, including Turkey (Ozturk & Ucsular 2011), India (Kirby & Cooper 2007), United States (Kirby et al. 2016b) and Canada (Best et al. 2005; MacPhee et al. 2004). A recent systematic review of 10 randomised controlled trials has confirmed the safety and effectiveness of wheelchair skills training (Tu et al. 2017). This trait of universal applicability in training tools is crucial for the global standardisation of wheelchair service provision education.

Respondents from educational institutions that did not currently teach wheelchair service content expressed an interest to do so. With this group of respondents, we saw the opportunity to show them the available resources, specifically the WHO WSTP, through a series of questions. Before participating in this survey study, less than half of this respondent group were aware of the WHO WSTP, but the majority indicated an interest in integrating the WHO WSTP into their programme curricula. This interest also aligns with students’ enthusiasm in wheelchair education as demonstrated in previous studies (Giesbrecht et al. 2015; Kirby et al. 2011). In these studies, students volunteered to attend wheelchair skills testing and training workshops (based on the Wheelchair Skills Program) offered on an extracurricular basis without promise of credit (Giesbrecht et al. 2015; Kirby et al. 2011). This voluntary choice may reflect the importance of wheelchair service provision education as perceived by students in health professional programmes. Five of the 12 (41.7%) educational institutions interested in teaching wheelchair content have already reached out to begin the development of a wheelchair service provision course. This finding suggests an opportunity for ISWP to initiate partnerships for the integration of wheelchair service provision education.

The inconsistency found in current wheelchair service provision education highlights an opportunity to integrate all WHO eight steps of wheelchair service provision. Our study found that some educational institutions acknowledged this need for a universal programme with flexibility to adapt a variety of considerations. One consideration is the physical environment, for example the type of cushion material needs to be suitable for the local climate. Another aspect to consider is the scope of practice of different rehabilitation professionals. For example, programmes such as OTA reported offering wheelchair-related education, but highlighted that only parts of the WHO eight-step model applied to the scope of OTA. On the other hand, in LRSs where access to rehabilitation service is a challenge (World Health Organization 2011), any health workers trained in wheelchair provision would increase appropriate wheelchair service provision. The emergence of community-based rehabilitation training presents an opportunity to explore training non-rehabilitation professionals who can then assist in wheelchair provision (Seymour 2016).

## Limitations

This study had several limitations. The volunteer sample captured using this cross-sectional research design may have consisted of individuals who prioritise and had pre-existing interest in wheelchair service provision education. Thus, the results cannot be generalised to all educational institutions that may or may not include wheelchair service provision education. Additionally, the sample was underrepresented in respondents from LRSs. As the survey was Internet-based and written in English, these factors may have limited the participation to respondents who were comfortable responding in English. Finally, each respondent answered on behalf of his or her entire institution, possibly masking the differences between each professional programme offered.

## Future studies

Future studies need to address the limitations by including translated, low-bandwidth and paper options to reduce bias in the recruitment. A follow-up survey will further investigate the topics in original wheelchair service provision education to see if and how they reflect the WHO eight-step model. Additional detail on wheelchair service provision content in curricula specific to each professional programme will be collected directly from stakeholders in academia who participate in the development of curricula, such as programme directors. Information on pedagogic methods (e.g. in class lectures or distance education programmes) of current and prospective wheelchair service provision content will also enlighten the situation. Other initiatives include qualitative interviews and partnerships with pilot sites that will enhance the ISWP’s understanding of barriers and facilitators faced by educational institutions currently integrating the WHO eight-step model into their curricula. Despite the limitations, this study is the first to describe current wheelchair service provision education in professional rehabilitation programme curricula on a global scale.

## Conclusion

Although the majority of the educational institutions reported teaching wheelchair-related content, there is great variability in what and how it is taught and evaluated. The WHO eight-step model and other readily available resources could serve as guides for wheelchair service provision education. The survey results inform the development of integration tools to guide educational curricula development, with the ultimate goal of improving the quality of wheelchair service provision worldwide.
